# Genome-associations of extended-spectrum ß-lactamase producing (ESBL) or AmpC producing *E. coli* in small and medium pig farms from Khon Kaen province, Thailand

**DOI:** 10.1186/s12866-022-02646-3

**Published:** 2022-10-20

**Authors:** João Pires, Laura Huber, Rachel A. Hickman, Simon Dellicour, Kamonwan Lunha, Thongpan Leangapichart, Jatesada Jiwakanon, Ulf Magnusson, Marianne Sunde, Josef D. Järhult, Thomas P. Van Boeckel

**Affiliations:** 1 Health Geography and Policy Group, Institute for Environmental Decisions, ETH Zurich, Zurich, Switzerland; 2 Department of Pathobiology, College of Veterinary Medicine, Auburn University, Auburn, AL USA; 3 Department of Medical Sciences, Uppsala University, Uppsala, Sweden; 4 Spatial Epidemiology Lab (SpELL), Université Libre de Bruxelles, Brussels, Belgium; 5 Department of Microbiology, Immunology and Transplantation, KU Leuven, Rega Institute, Louvain, Belgium; 6 Department of Clinical Sciences, Swedish University of Agricultural Sciences, Uppsala, Sweden; 7 Section for Food Safety and AMR, Norwegian Veterinary Institute, Oslo, Norway; 8 Research Group Preventive Technology Livestock, Khon Kaen University, Khon Kaen, Thailand; 9 Center for Diseases Dynamics Economics & Policy, Washington, DC USA

**Keywords:** Antimicrobial resistance, Pig, Farms, Antimicrobial use, Escherichia coli, ESBL

## Abstract

Thailand is undergoing rapid intensification of livestock production where small subsistence farms and medium sized commercial farms coexist. In medium farms, antimicrobials are prescribed by a veterinarian, whereas in small farms antimicrobial use remains largely unsupervised. The impact of these differences as well as other farming practices on the emergence and composition of antimicrobial resistance genes (ARGs) remains largely unknown. We analyzed 363 genomes of extended-spectrum ß-lactamase producing (ESBL) and/or AmpC producing *Escherichia coli* recovered from humans and pigs at small and medium farms from the Khon Kaen province, Thailand. We tested for genome-wide associations to identify links between ARGs, host, and farm size. Pig isolates from small farms were associated with *mcr* and *qnr* genes conferring resistance to colistin and fluoroquinolones, respectively. In contrast, pig isolates from medium farms were associated with ARGs conferring resistance to drugs commonly used on medium farms (i.e., streptomycin). ESBL plasmids from small farms co-carried ARGs conferring resistance to critically important antimicrobials more frequently compared to plasmid from medium farms. Frequent ARG combinations included *bla*_CTX-M-55_ + *qnrS1* (29.8% vs 17.5% in small and medium farms, respectively), *bla*_CTX-M-55_ + *qnrS1* + *mcr-3.19* (5% vs 0%), *bla*_CTX-M-14_ + *qnrS1* (9.3% vs 6.2%), and *bla*_CTX-M-14_ + *qnrS1* + *mcr-1.1* (3.1% vs 0%). The co-location on plasmids of ARGs conferring resistance to critically important antimicrobials as defined by the World Health Organization is concerning, and actions to curb their spread are urgently needed. Legislation on limiting antimicrobial sales and initiatives to better inform farmers and veterinarians on appropriate antimicrobial usage and farm biosecurity could help reduce antimicrobial use on farms.

**Supplementary Information:**

The online version contains supplementary material available at 10.1186/s12866-022-02646-3.

## Introduction

Antimicrobial resistance (AMR) is a global threat that has been driven by the overuse of antimicrobials in humans and animals [1]. Antimicrobial consumption in animals is increasing globally and represents 73% of the global antimicrobial sales [2, 3]. The increase in antimicrobial use (AMU) in animals is driven by the global increase in demand for animal protein which led to the intensification of animal-production, particularly in low- and middle-income countries (LMICs) [3–5]. In countries undergoing this rapid intensification, it has been suggested that, antimicrobials are used as growth promoters and as surrogates for adequate hygiene measures and good farming practices [6], which has locally lead to increase in AMR levels in animals [7, 8]. In high-income countries, works have shown that organic farms have lower AMR levels compared to conventional farms [9–12]. In addition, increased farm biosecurity and animal welfare has also shown to have impacts on AMU, in turn, affecting AMR levels [13–16]. Understanding the contribution of farming practices on AMR levels is thus instrumental for future AMR management but still insufficiently understood.

The Thai pig-production system provides a unique opportunity to study the effects of farming-practices on AMU and on transmission of AMR bacteria for multiple reasons. First, as in many LMICs, antimicrobials can be bought over-the-counter and administered without consulting a veterinarian [17]. Second, farms at different stages of intensification co-exist in close geographic proximity [18]. As biosecurity and AMU standards differ between the extensive and intensive farms in Thailand [19], bacteria face different selective pressures that may influence their ability for accessory gene acquisition, including antimicrobial resistance genes (ARGs) [20]. Moreover, different biosecurity levels may also affect the transfer of AMR bacteria between animals and farmers [21], including Extended-spectrum-ß-lactamase (ESBL)- or AmpC-producing *Escherichia coli* [22]*.*

To date, there is diverse information on the transmission of ESBL- or AmpC-producing *E. coli* between humans and food-producing animals [23, 24]. On the one hand, the same sequence types (ST) and ß-lactamase types have been identified in both humans and animals, indicating a degree of sharing of these bacteria between hosts [23, 25]. Furthermore, in farmers, occupational exposure to the animals on-farm has been suggested as is a risk factor for ESBL-colonization [26]. On the other hand, clonal transmission of ESBL has rarely been reported [27], and works have shown that the strains circulating in livestock are genetically distinct from those circulating in humans [28]. These observations could indicate two things: first that the temporality of the transmission of ESBL and/or AmpC producing *E. coli* occurs at scales that cannot be observed through cross-sectional studies. Second, that transmission is mediated via horizontal gene transfer of plasmids or other mobile genetic elements (MGEs) where ESBLs or AmpCs are commonly located [29, 30]. Multiple works have used core-genome to assess transmission ESBL/AmpC-transmission. However, MGE information is often discarded as they are commonly classified as part of the accessory genome [31]. Therefore, exploring this accessory genome could help understand the effect of farming practices on the composition of the accessory genome of bacteria, as well as provide insight into potential transmission events between different hosts [30].

In a previous study, we show that ESBL-producing *E. coli* were highly prevalent in both small and medium pig farms and that pigs and humans shared the same sequence types [32]. The transmission of these resistant microorganisms between pigs and farmers was hypothesized but not confirmed [32, 33]. Here, we investigate the impact of farm size and host on the accessory-genome of ESBL and/or AmpC-producing *E. coli* recovered from pigs and humans and investigate the inter-host transmission of these microorganisms using whole genome sequencing.

## Materials and methods

### Bacterial genomes

We retrieved 363 ESBL and/or AmpC-producing *E. coli* from a study performed across 155 farms Khon Kaen province of Northeast Thailand from September to December, 2018 [32]. Isolates were recovered from fecal swabs of healthy pigs, pig-farmers (human contacts), and people living in the same household of pig-farmers but not in contact with the farm environment (human non-contacts). Swabs were enriched and plated onto antibiotic-selection plates to recover ESBL and/or AmpC-producing isolates. Different morphotypes were picked and identified by Matrix-assisted laser desorption ionization time of flight (MALDI-TOF). All identified *E. coli* underwent whole-genome sequencing and the presence of ESBL and/or AmpC genes was confirmed by ResFinder [34]. A full description of the study design, bacterial isolation, characterization, and DNA extraction and whole genome sequencing can be found in Hickman et al*.* [32]. A total of 237 (65.3%) genomes were recovered from small-size farms of which 120 (50.6%) were recovered from pigs, 75 (31.6%) from contacts, and 42 (17.7%) from non-contacts (Table 1). The remaining 126 genomes (34.7%) were recovered from medium-size farms, of which 68 (54%) were recovered from pigs, 40 (31.7%) from contacts, and 18 (14.3%) from non-contacts (Table 1). All genomes are available in the European Nucleotide Archive BioProject PRJEB38313 (Table S1).

**Table 1:** Distribution of farm size, host and ß-lactamase gene in ESBL and/or AmpC-producing *E. coli*

Farm Size	Host	ß-Lactamase Gene^a^					
		*bla*_CTX-M-55_	*bla*_CTX-M-14_	*bla*_CTX-M-27_	*bla*_CTX-M-15_	*bla*_CMY-2_	Other ESBL
Small (*n* = 237)	Pig (*n* = 120, 50.6%)	77 (64.17%)	34 (28.33%)	-	3 (2.5%)	4 (3.33%)	2 (1.67%)
	Human Contact (*n* = 75, 31.6%)	33 (42.86%)	26 (33.77%)	6 (7.79%)	7 (9.09%)	1 (1.3%)	4 (5.19%)
	Human Non-Contact (*n* = 42, 17.7%)	14 (33.33%)	11 (26.19%)	6 (14.29%)	8 (19.05%)	3 (7.14%)	-
Medium (*n* = 126)	Pig (*n* = 68, 54.0%)	21 (30.43%)	28 (40.58%)	-	-	17 (24.64%)	3 (4.35%)
	Human Contact (*n* = 40, 31.8%)	15 (34.88%)	12 (27.91%)	6 (13.95%)	5 (11.63%)	4 (9.3%)	1 (2.33%)
	Human Non-Contact (*n* = 18, 14.3%)	10 (52.63%)	2 (10.53%)	4 (21.05%)	-	3 (15.79%)	-

^a^Only ß-lactamases that confer resistance to extended-spectrum ß-lactams are shown

### Bioinformatic analysis

**Fig. 1:**
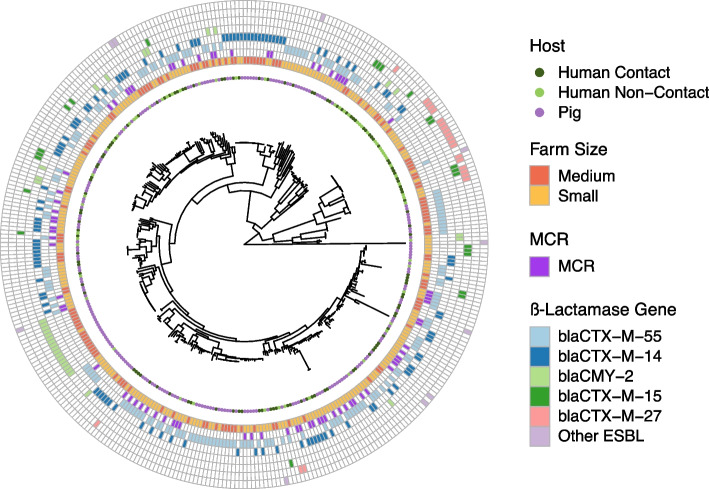
Phylogenetic tree obtained from core-genome SNPs of pig and human isolates from both small and medium farms

All assemblies were annotated with Bakta (version 1.2.1) [35] and a core-genome alignment was obtained using Roary (version 3.13.0) [36]. Single Nucleotide Polymorphisms (SNPs) in the core-genome were retrieved using SNP-sites (version 2.5.1) [37], and the resulting output was used to construct a phylogenetic tree with 1,000 bootstrap replicates using IQ-TREE (version 8.2.4) with the general time reversible model, with gamma rate variation among sites and empirical codon frequencies from the data (GTR + F + G4) [38]. The phylogenetic tree was imported and visualized using the R (version 4.1.1) package “ggtree” [39]. Acquired antimicrobial resistance genes (ARGs) were identified using ABRicate [40] (version 1.0.1) with the ResFinder database (database version of March 5^th^, 2021) [34]. Only genes with a 90% identity and coverage were kept. Figure 1 summarizes the distribution of the isolates’ host and farm-size, as well as the carriage of ESBL, AmpC and *mcr* genes along the core-genome phylogeny. Plasmid contigs were grouped into distinct plasmid clusters per genome using MOB-suite with the mob-recon module (version 3.0.3) [41]. Contigs containing ARGs were matched to those classified as plasmids.

### On-Farm transmission analysis

We calculated the MASH pairwise distances between whole-genomes using Pangenome Analysis Toolkit (PATO) package [42]. We then calculated whole-genome average nucleotide identity (ANI) as follows: ANI = 1 – Distance_MASH_ × 100. On-farm transmission between pigs and humans or between human-contacts and non-contacts was considered when pairs of isolates had an ANI above 99.9%. This threshold enables to distinguish closely related strains and is above the threshold to identify sub-groups within *E. coli* phylogenetic groups (MASH distance 0.0185, ANI of ~ 98.85%) [43, 44]. Clonal transmission between-farms was not considered since we do not have data to support evidence of between-farm transfer (e.g., epidemiological information, transport of pigs between farms, or farmers visiting and/or working in multiple farms). In addition, isolate pairs found in the same farm and host were not considered since information on whether samples were isolated from different farmers or pigs.

### Core-Genome phylogenetic analysis

We used the core-genome to understand the effects of different covariates on the genetic distance and phylogeny of our sample. First, we tested for the presence of a spatial autocorrelation in our genomes by calculating a semi-variogram (R package “phylin” version 2.0.2, [45]). The genetic distance was obtained from the core-genome SNP-alignment (snp-sites version 2.5.1, [46]) using the “dna.dist” function from the R package “ape” [47], and the physical distance was calculated using the isolates’ geographic-coordinates. Finally, we used a Mantel test to assess the correlation between the genetic and the geographical distances with the “mantel” function from the “vegan” package (version 2.5.7 [48]). Thereafter, we investigated the correlation between the genetic distance with the host and the farm-size using a PERMANOVA ("adonis” function from the R package “vegan”).

Finally, we used our core-genome phylogenetic tree to assess the phylogenetic signal associated with different covariates (host, farm-size, antimicrobial use, antimicrobials used in the last month, used of antimicrobial supplemented feed in the last month, minimal distance to drug stores, presence of diseases), i.e. the tendency for phylogenetically close samples to share similar covariate values. Phylogenetic signal for discrete binary variables was tested with the *D* statistic estimated with the “phylo.d” function from the “caper” R package version 1.0.1 [49], and phylogenetic signal for continuous variables was tested with the Pagel’s λ and Blomberg *K* statistics estimated with the “phylosig” function from the “phytools” R package version 1.0.1 [50]. All tests were performed with 1,000 bootstraps.

### Plasmid similarity analysis

We identified plasmid clusters carrying the most frequent ESBLs and/or AmpC genes (*bla*_CTX-M-55_, *bla*_CTX-M-14_, *bla*_CTX-M-15_, *bla*_CTX-M-27_, and *bla*_CMY-2_) and extracted their contigs. We calculated the pairwise MASH distance across all plasmid cluster contigs carrying the same ESBL or AmpC using the “mash” function within the R package “PATO” [42, 51]. The obtained distance matrices were used to create UPGMA trees visualized with the R package “ggtree” [39]. Plasmid were considered identical when the pairwise ANI was above 99.9%. Plasmid similarity was only considered when identical plasmids were found between different STs. We compared the physical distance of isolates sharing plasmids to those not sharing plasmids using a t-test (“t.test” function from the “stats” R package version 4.1.1.).

### Statistical analysis of accessory genome

We calculated statistical associations between genes and their host and/or farm-sizes using genome-wide associations (GWAS) with Scoary (version 1.6.16, [52]). We performed the analysis using the gene presence-absence matrix from Roary as input and accounted for the population structure by feeding the phylogenetic tree. This analysis was bootstrapped 1,000 times. Only genes with both a Bonferroni adjusted *p*-value and empirical *p*-value below 0.05 were kept. Furthermore, we used the Accessory Genome Constellation Network (AcCNET) module within the R package “PATO” to extract the accessory genome and create a bipartite network showing genomes that share a protein [42]. The resulting accessory-genome presence-absence matrix was used to create a Jaccard distance matrix with the R package “vegan” version 2.5.7, [48]. Thereafter, we tested whether the composition in the accessory genome differed between hosts and farm-sizes using a PERMANOVA. We used the enrichment analysis function within PATO to identify genes in the accessory genome overrepresented in different host and farm sizes. Only edges with an adjusted *p*-value below 0.05 were considered.

## Results

### Infrequent animal-human transmission on farms

Based on pairwise MASH distance calculations between whole genomes, we identified 244 pairwise comparisons with an ANI above 99.9%. This indicated that these isolate pairs could be considered the same bacterial clone and thus potentially be involved in transmission events between humans and animals or between contacts and non-contacts. Only 10 isolate pairs (8 events) corresponded to suspected on-farm transmission events between hosts (Table 1). Six suspected transfer events were identified in small farms and four in medium farms. In small farms, three of the transfers suspected to have occurred between pigs and humans. In contrast, this was only observed once in medium farms. While in medium farms only ESBLs or AmpCs were involved, four suspected transmission events in small farms included isolates that also co-carried *mcr* genes (Table 2).

**Table 2:** Characteristics of isolates involved in within-farm transmission

Event	Isolate ID	Farm Number	Farm Size	Host	PhG^a^	ST^b^	ESBL/AmpC	ARGs^c^
I	D103CM1	103	small	Human Contact	B1	278	*bla*_CTX-M-55_	*aac(3)-IId, bla*_TEM-1B_*, qnrS1, tet(A), catA2, mcr-3.19*
	D103UM1	103	small	Human Non-Contact	B1	278	*bla*_CTX-M-55_	*aac(3)-IId, bla*_TEM-1B_*, qnrS1, tet(A), catA2, mcr-3.19*
II	D120CM2	120	medium	Human Contact	A	6786	*bla*_CTX-M-55_	*aac(3)-IId, bla*_TEM-1B_*, dfrA12, mef(B), qnrS1, sul3, tet(A), lnu(F)*
	D120PM3	120	medium	Pig	A	6786	*bla*_CTX-M-55_	*aac(3)-IId, dfrA12, qnrS1, sul3, tet(A), lnu(F), erm(B)*
III	D123CM1	123	medium	Human Contact	B1	58	*bla*_CMY-2_ , *bla*_CTX-M-55_	*aph(3'')-Ib, aph(6)-Id, bla*_TEM-1B_*, qnrS1, tet(A), aph(3')-Ia, floR, sul2, dfrA14, aac(3)-IIa*
	D123UM1	123	medium	Human Non-Contact	B1	58	*bla*_CMY-2_ , *bla*_CTX-M-55_	*aph(3'')-Ib, aph(6)-Id, bla*_TEM-1B_*, qnrS1, tet(A), aph(3')-Ia, floR, sul2, dfrA14, aac(3)-IIa*
IV	D126CM1	126	medium	Human Contact	D	38	*bla*_CTX-M-55_	*ant(3'')-Ia, aph(6)-Id, tet(A), aph(3')-Ia, floR, catA2, aac(3)-IIa, bla*_TEM-215_
	D126UM1	126	medium	Human Non-Contact	D	38	*bla*_CTX-M-55_	*ant(3'')-Ia, aph(6)-Id, tet(A), aph(3')-Ia, floR, catA2, aac(3)-IIa, bla*_TEM-215_
V	D43PM1	43	small	Pig	A	10	*bla*_CTX-M-55_	*qnrS1, aph(3')-Ia, tet(X4), catA2, bla*_TEM-176_
	D43UM1	43	small	Human Non-Contact	A	10	*bla*_CTX-M-55_	*qnrS1, aph(3')-Ia, tet(X4), catA2, bla*_TEM-176_
VI	D63PM2	63	medium	Pig	F	457	*bla*_CTX-M-55_	*sul3, tet(A), aph(3')-Ia, floR, lnu(F), dfrA14, aac(3)-IIa*
	D63UM2	63	medium	Human Non-Contact	F	457	*bla*_CTX-M-55_	*sul3, tet(A), aph(3')-Ia, floR, lnu(F), dfrA14, aac(3)-IIa*
VII	D95CM1	95	small	Human Contact	B1	515	*bla*_CTX-M-14_	*aac(3)-IId, aadA2, ant(3'')-Ia, aph(3'')-Ib, aph(6)-Id, bla*_TEM-1B_*, cmlA1, dfrA12, mef(B), qnrS1, sul3, tet(A), aph(3')-Ia, mcr-1.1*
	D95PM1	95	small	Pig	B1	515	*bla*_CTX-M-14_	*aac(3)-IId, aadA2, ant(3'')-Ia, aph(3'')-Ib, aph(6)-Id, bla*_TEM-1B_*, cmlA1, dfrA12, mef(B), qnrS1, sul3, tet(A), lnu(F)*
	D95UM2	95	small	Human Non-Contact	B1	515	*bla*_CTX-M-14_	*aac(3)-IId, aadA2, ant(3'')-Ia, aph(3'')-Ib, aph(6)-Id, bla*_TEM-1B_*, cmlA1, dfrA12, mef(B), qnrS1, sul3, tet(A), aph(3')-Ia, mcr-1.1*
VIII	D98CM1	98	small	Human Contact	A	10,562	*bla*_CTX-M-14_	*aac(3)-IId, aadA2, ant(3'')-Ia, aph(3'')-Ib, aph(6)-Id, bla*_TEM-1B_*, cmlA1, dfrA12, mef(B), qnrS1, sul3, aph(3')-Ia, mcr-1.1, tet(B), mcr-3.1*
	D98UM1	98	small	Human Non-Contact	A	10,562	*bla*_CTX-M-14_	*aac(3)-IId, aadA2, ant(3'')-Ia, aph(3'')-Ib, aph(6)-Id, bla*_TEM-1B_*, cmlA1, dfrA12, mef(B), qnrS1, sul3, aph(3')-Ia, mcr-1.1, tet(B), mcr-3.1*

^a^*E. coli* phylogenetic group

^b^Sequence type

^c^Antimicrobial resistance gene

### Farming practices influence core-genome phylogeny

The genetic distance of genomes was not associated with an autocorrelative spatial structure based on the genetic semi-variogram or the Mantel test (*p* = 0.997). However, the core-genome genetic distance was significantly associated with the farm type (*p* = 0.003) and host (*p* = 0.001).

We also identified a supported phylogenetic signal with the *D* statistic for host (*D* = 0.692, *p* < 0.001) and farm-size (*D* = 0.532, *p* < 0.001). Phylogenetic signal was also observed for the use of antibiotic supplemented feed (*D* = 0.598, *p* < 0.0001) and antimicrobial use in the last month (*D* = 0.892, *p* = 0.043). For the continuous variable antimicrobial use we detected a low yet significant phylogenetic signal (Blombergs *K* = 0.0003, *p* = 0.001; Pagel’s λ = 0.145, *p* = 0.015).

### Limited evidence for plasmid sharing between hosts and/or farms

**Fig. 2:**
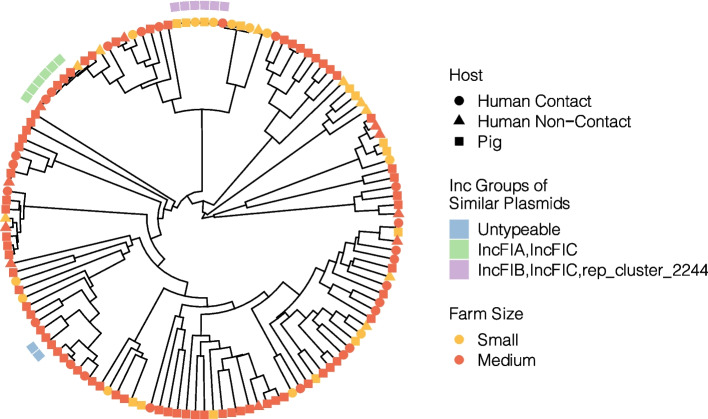
UPGMA tree based on MASH distance of plasmids carrying *bla*_CTX-M-55_. Outer ring boxes mark plasmids that have an Average Nucleotide Identity above 99.9%

**Fig. 3:**
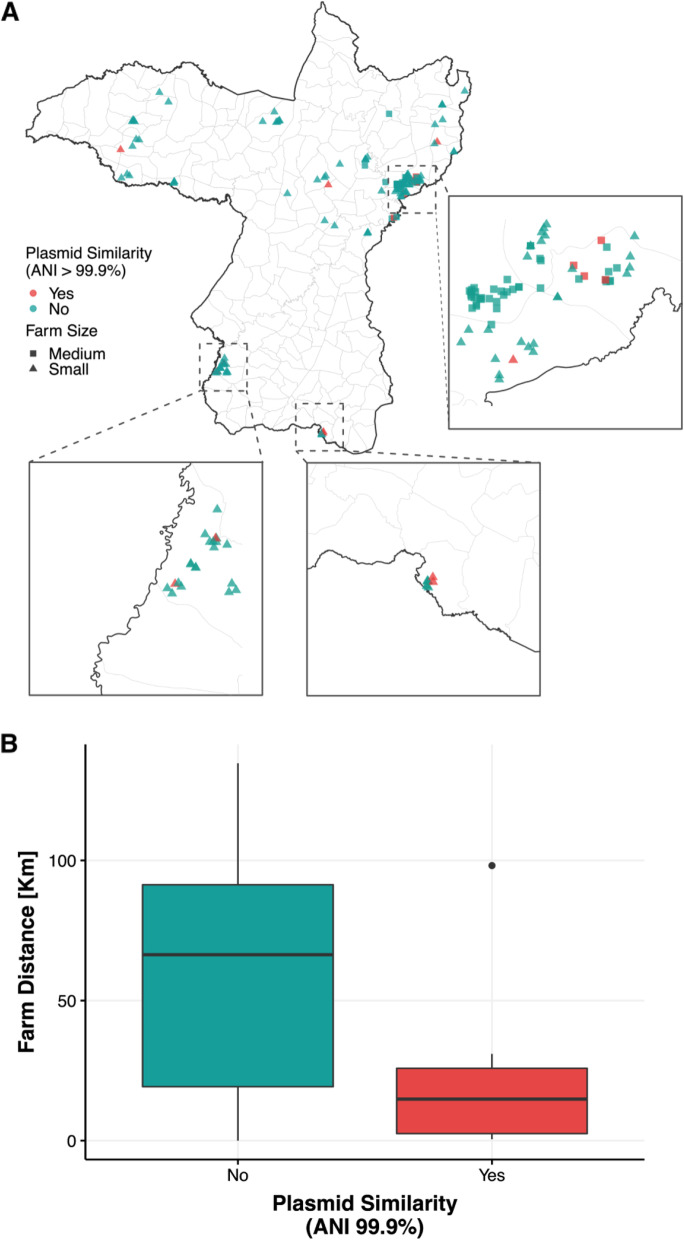
**A** Pig farms in Khon Kaen province, Thailand. Red indicates that a similar plasmid carrying *bla*_CTX-M-55_ was found in a different farm, green indicates farms where no similar *bla*_CTX-M-55_-carrying plasmid was found; **B** Boxplots with the farm-distance between similar plasmids (Average Nucleotide Identity > 99.9%) versus those not involved in transmission

Based on the pairwise MASH distance between plasmid cluster contigs containing ESBL and/or AmpC, suspected plasmid sharing was only observed for plasmids carrying *bla*_CTX-M-55_. A total of four plasmid-sharing groups were identified (Fig. 2, Table 3). Group I (*n* = 6) contained five isolates from medium-sized farms (three of pig and two of human origin, respectively) and one from a small farm (human). This plasmid is characterized by an IncFIB-FIC-rep_cluster_2244 plasmid that co-carries several aminoglycoside modifying enzymes, *bla*_TEM-1B_ and *sul2*. Group II (*n* = 4) included only isolates from small farms (3 from human origin and one from pig). The plasmid shared among this group is an IncFIA-FIC co-carrying *aac(3)-IId, mcr-3.19,* and *qnrS1.* Groups III and IV comprised of pig isolates from small farms of two isolates each. Group III consists of a IncFIA-FIC carrying also *qnrS1*, whereas Group 4 comprises a small plasmid fragment where no without a *rep* genes and no other resistance gene is predicted to be present on this plasmid. Isolates that shared the same plasmid were geographically clustered compared to isolates that did not share the same plasmid (Fischer test *p* = 0.003; Fig. 3).

**Table 3:** Characteristics of shared plasmids carrying *bla*_CTX-M-55_

Event	Isolate ID	Farm Number	Farm Size	Host	ST	PhG	Size (bp)^a^	Incompatibility Group^b^	ESBL	Plasmid ARG^c^
I	D108CM2	108	Small	Human Contact	354	F	113,076	IncFIB, IncFIC, rep_cluster_2244	*bla*_CTX-M-55_	*aph(3'')-Ib, aph(3')-Ia, aph(6)-Id, bla*_TEM-1B_*, dfrA14, sul2*
	D128CM1	128	Medium	Human Contact	10	A	115,529	IncFIB, IncFIC, rep_cluster_2244	*bla*_CTX-M-55_	*aph(3'')-Ib, aph(3')-Ia, aph(6)-Id, bla*_TEM-1B_*, sul2*
	D132PM1	132	Medium	Pig	354	F	115,584	IncFIB, IncFIC, rep_cluster_2244	*bla*_CTX-M-55_	*aph(3'')-Ib, aph(3')-Ia, aph(6)-Id, dfrA14, sul2*
	D133PM1	133	Medium	Pig	354	F	116,447	IncFIB, IncFIC, rep_cluster_2244	*bla*_CTX-M-55_	*aph(3'')-Ib, aph(3')-Ia, aph(6)-Id, bla*_TEM-1B_*, dfrA14, sul2*
	D136PM3	136	Medium	Pig	354	F	116,447	IncFIB, IncFIC, rep_cluster_2244	*bla*_CTX-M-55_	*aph(3'')-Ib, aph(3')-Ia, aph(6)-Id, bla*_*TEM-1B*_*, dfrA14, sul2*
	D163CM1	163	Medium	Human Contact	58	B1	113,577	IncFIB, IncFIC, rep_cluster_2244	*bla*_CTX-M-55_	*aph(3'')-Ib, aph(3')-Ia, aph(6)-Id, bla*_TEM-1B_*, sul2*
II	D103CM1	103	Small	Human Contact	278	B1	85,115	IncFIA, IncFIC	*bla*_CTX-M-55_	*aac(3)-IId, mcr-3.19, qnrS1*
	D18CM1	18	Small	Human Contact	155	B1	86,116	IncFIA, IncFIC	*bla*_CTX-M-55_	*aac(3)-IId, mcr-3.19, qnrS1*
	D103UM1	103	Small	Human Non-Contact	278	B1	85,115	IncFIA, IncFIC	*bla*_CTX-M-55_	*aac(3)-IId, mcr-3.19, qnrS1*
	D35PM1	35	Small	Pig	10	A	82,607	IncFIA, IncFIC	*bla*_CTX-M-55_	*aac(3)-IId, mcr-3.19, qnrS1*
III	D75PM1	75	Small	Pig	101	B1	75,515	IncFIA, IncFIC	*bla*_CTX-M-55_	*qnrS1*
	D76PM1	76	Small	Pig	6449	A	77,402	IncFIA, IncFIC	*bla*_CTX-M-55_	*qnrS1*
IV	D141PM1	141	Small	Pig	10,587	E	1,586	-	*bla*_CTX-M-55_	-
	D49PM2	49	Small	Pig	10,578	A	1,586	-	*bla*_CTX-M-55_	-

^a^Plasmid size according to the contigs assigned to the different plasmids by MOB-suite

^b^Incompatibility group retrieved from MOB-suite

^c^Antimicrobial Resistance Genes co-located on the same plasmid

### High co-occurrence of ESBL or AmpC genes with ARGs conferring resistance to critically important antimicrobials

**Fig. 4:**
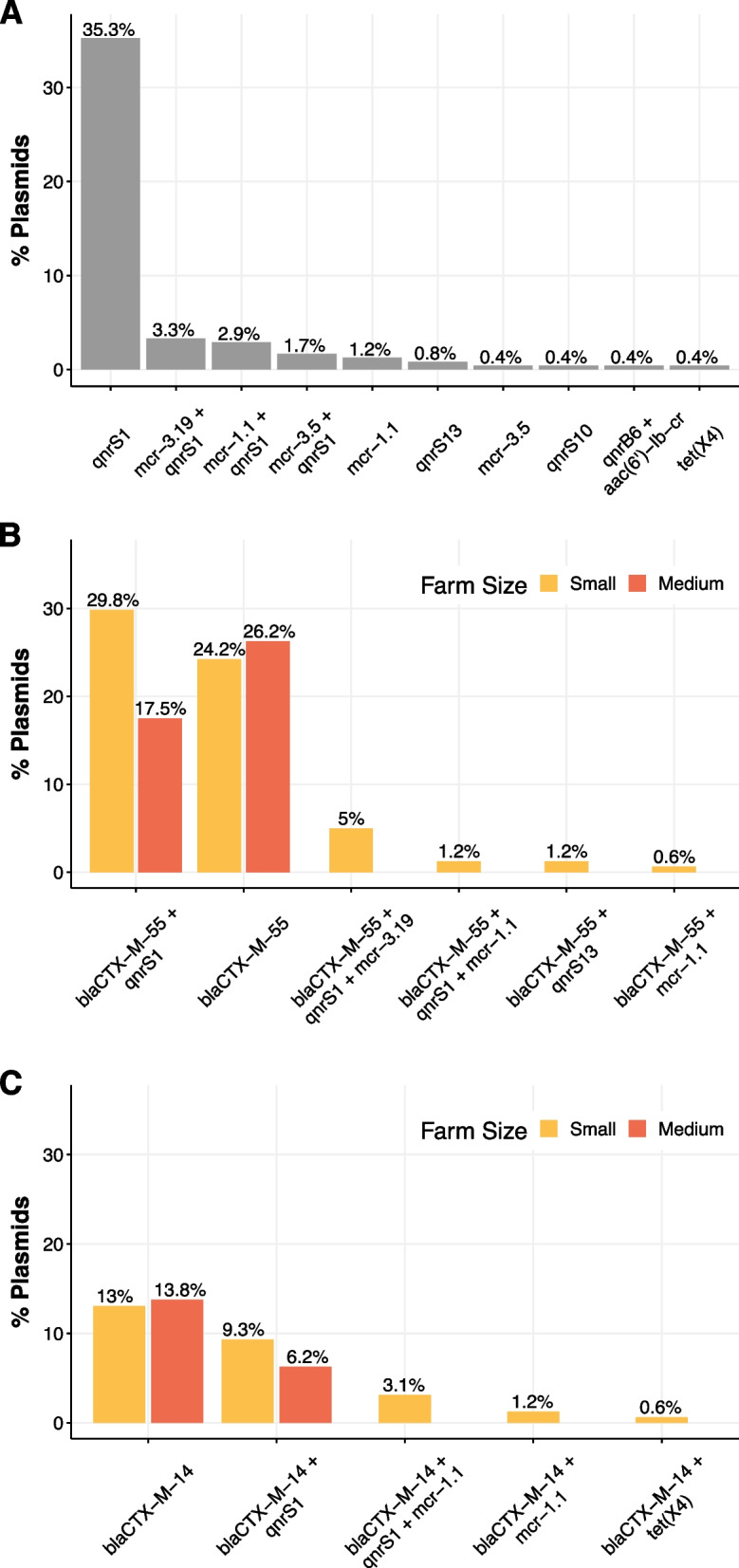
**A** Proportion of ESBL and/or AmpC-carrying plasmids (*n* = 241) that co-carry resistance to critically important antimicrobials. **B** Proportion of *bla*_CTX-M-55_ carrying plasmids that co-carry resistance to critically important antimicrobials in small and medium farms. **C** Proportion of *bla*_CTX-M-14_ carrying plasmids that co-carry resistance to critically important antimicrobials in small and medium farms. Proportions in panels B and C are calculated based on the total ESBL and/or AmpC plasmids in small (*n* = 161) and medium (*n* = 80) farms

Of all ESBL and/or AmpC-carrying plasmids (*n* = 241), nearly half (*n* = 114, 47.3%; Fig. 4) also co-carried genes conferring resistance to critically important antimicrobials as defined by the World Health Organization (Fig. 4). Frequently, these plasmids co-carried *qnrS1* (*n* = 80, 35.3%), and to a lesser extent *mcr-3.19* plus *qnrS1* (3.3%), *mcr-1.1* plus *qnrS1* (2.9%), *mcr-3.5* plus *qnrS1* (1.7%), *mcr-1.1.* (1.1%), among others. Co-carriage was mostly observed for plasmids carrying *bla*_CTX-M-55_ (Fig. 4B) and *bla*_CTX-M-14_ (Fig. 4C). One *bla*_CMY-2_-carrying plasmids also harbored the ESBL *bla*_TEM-106_ and *qnrS10*; and a *bla*_CTX-M-15_-carrying plasmid also co-carried *qnrB6* and *aac(6’)-Ib-cr*. *mcr* genes were only present in plasmids from small farms. Moreover, plasmids from small farms more frequently co-carried genes conferring resistance to critically important antimicrobials compared to medium farms (χ^2^ = 15.441, *p* = 8.514e^−5^).

### Host and farming practices influence accessory genome content and resistance gene content

Based on GWAS analysis, we did not identify differences in gene content between isolates from farmers and non-farmers. Therefore, these two categories were collapsed into a pooled category called ‘human’. When comparing GWAS based on the farm-size, we identified only one gene associated with medium-sized farms (Transposase IS66 family). For small farms, we identified 4 genes with small-farms: a diacylglycerol kinase, a periplasmic protein, the transposase *tnpA12* and the colistin resistance gene *mcr-3.5*. Thereafter, we investigated gene associations in the different combination of farm-size and host. No gene was associated with human isolates from either farm-sizes. Four genes were associated with pigs from medium farms, and 3 genes with small-farms, including the *mcr-3.5* gene.

We further investigated the accessory genome composition with AcCNET. We obtained a network consisting of 361,517 elements with 18,692 unique proteins. The accessory genome content between differed between different host and farm sizes (PERMANOVA, both *p* < 0.001). An interaction between host and farm size was also identified (*p* < 0.001). A total of 3,285 genes were identified to be enriched in one of the labels, of which 1,017 in pigs from small farms, 967 in humans from small farms, 874 in pigs from medium farms, and 427 in humans from medium farms. Eleven resistance genes (0.33% of all genes) were also found to be enriched: four genes in pigs from small farms (*mcr-3.2*, *mcr-3.5*, *bla*_CTX-M-55_, and *qnrS13*); three in pigs from medium farms [*bla*_CMY-2_, *aac(3)-IId*, and *aph(3’)-Ia*]; three in humans from small farms [*tetC*, *aadA5*, *aph(3’)-Ia*]; and one in humans from medium farms (*dfrA7*).

## Discussion

Countries undergoing rapid intensification of animal production such as Thailand provide a unique opportunity to better understand the role of farming practices on the dissemination of AMR at the farm level [53, 54]. In 2019, a total of 2,566,704 kg (Mulchandani et al., unpublished results) of antimicrobials were sold in Thailand for animal use. Understanding the impact of AMU in different contexts is of extreme importance to identify *novel* ways to control the spread of AMR. In this study, we show important differences in the resistance gene content in ESBL and/or AmpC-producing *E. coli* recovered from small farms compared to medium farms in the Khon Kaen province, Thailand.

We found potential evidence of a total of 8 events of on-farm transmission (Table 2). Among these, four of them involved human and pig isolates, two from each farm size, indicating that occupational exposure in either farm size is a potential route of acquisition of multidrug resistant bacteria. These events were rare in our study, which is consistent with the current literature that only reported few clonal transmissions events [26, 55]. Despite this, these transmissions should not be neglected, especially given that some of these isolates co-carry other antimicrobial resistance genes conferring resistance to critically important antimicrobials.

We further investigated the impact of different covariates on the grouping of isolates at the tips of the tree by testing the phylogenetic signal. Our analysis indicates that farm size, host category and the use of antibiotic supplemented feed do not follow a random distribution across the phylogenetic tree (*D* value < 1). This could potentially be explained by the fact that the same STs are found in the same farms or hosts. However, the lack of spatial autocorrelation in our dataset could suggest that the isolates of our study are randomly distributed in the region. Therefore, this implies that the different hosts and farming practices have some degree of influence in our isolates which will likely reflect on the accessory gene composition. This has been supported by GWAS and the AcCNET enrichment analysis of the accessory genome. Overall, small-scale farms are significantly associated with ARGs that confer resistance to last resort antimicrobials including colistin (*mcr* genes) and ciprofloxacin (*qnr* genes), whereas medium farms were enriched in genes associated with streptomycin resistance (*aac(3)-IId*, and *aph(3’)-Ia*). The combination of *mcr* genes with *bla*_CTX-M-55_ has also been found in other Thai regions and countries in Southeast Asia [56]. The presence of these genes is consistent with the most commonly used antimicrobials in the different farm sizes. In medium farms, the most common antimicrobials used were Penicillin G-Streptomycin combinations (*n* = 49, 100%, Table S2). In small farms reporting this information (*n* = 65, 61.3%), the most frequent antimicrobials used were enrofloxacin (*n* = 26, 40%), Penicillin G-Streptomycin combinations (*n* = 15, 23.1%), amoxicillin (*n* = 8, 12.3%) and oxytetracycline (*n* = 7, 10.8%). From a previous epidemiological study in the same region, medium-scale farms show higher antimicrobial use compared to small-farms [33]. However, in medium farms, antimicrobials are administered under the supervision of veterinarians, whereas small-scale farms typically get advice from drug stores [33]. Among the 67 small-scale farms that reported AMU, enrofloxacin was the most frequent antimicrobial used (38.8%) [33], which would explain the enrichment of *qnrS* genes herein. While farms in our study did not report colistin use, we need to consider that not all farms using antimicrobials reported which were administered and the potential influence of recall-bias. However, a previous studies in Thai pig farms identified that farms using colistin were also associated with *mcr* genes [18, 57], as observed elsewhere [58]. Furthermore, a previous study in Thailand show that a majority of antimicrobials used on farms were critically important antimicrobials including colistin and enrofloxacin [17]. This suggests that lower but unregulated and unsupervised use of critically important antimicrobials selects for ARGs conferring resistance to these antimicrobials. Unsupervised use of antimicrobials in small-scale farms leading to high AMR levels has also been observed in the neighboring country of Cambodia [59].

Previous works have shown that the carriage of ESBL and/or AmpC-producing bacteria is influenced by the farming practices (conventional, organic, or other antibiotic-free), biosecurity levels and antimicrobial use [14, 16, 55, 60, 61]. In these studies, higher levels of biosecurity and lower AMU were associated with lower levels of ESBL and/or AmpCs-carriage in pigs. However, the statistical association of other critically important ARG was yet to be reported. Our study, to the best of our knowledge, is the first to report at farm level, a statistical association of the level of farming intensification with genes conferring resistance to critically important antimicrobials in ESBL/AmpC producing *E. coli*.

The enrichment of ARGs conferring resistance to critical important antimicrobials in animals could also serve a source for further dissemination of these mechanisms to other hosts and/or farms. This is supported by our clonal and plasmid similarity analysis (Tables 1 and 2). In both cases, we identify clones and plasmids that contain both the ESBL/AmpC, *mcr* and *qnrS* genes in both humans and animals across farms. Plasmid transmission between pigs as well as between pigs and farmers has also been previously documented, although not frequently [29, 62]. The transmission routes are challenging to establish, but close proximity seems to be influencing plasmid transmission (Fig. 3) [63]. The acquisition of the same plasmids in different hosts and/or farms might suggest a common environmental compartment that is shared by different farms (e.g., water), other unknown indirect transmission routes [64] or plasmid transmission into the hosts’ bacterial flora during transient gut colonization [65]. An alternative explanation of plasmid sharing in multiple farms could be the movement of people and animals between farms colonized with these bacteria and/or plasmids, as observed for other microorganisms [66–68]. A recent study highlighting the Thai pig trade network indicated that this network could facilitate the spread of infectious diseases [69].

Finally, we investigated whether ARGs from the enrichment analysis were predicted to be on the same plasmids where the EBSL/AmpC gene was located. We identified that nearly 50% plasmids also harbored at least one other ARG conferring resistance to critically important antimicrobials. While we do not have access to the farming system, a Vietnamese study has also identified that several ESBL-carrying plasmids also co-carry ARGs conferring resistance to critically important antimicrobials [70]. Additionally, we identified that plasmids from small farms were more likely to co-carry these ARGs than those from medium farms. This highlights that a lack of antimicrobial stewardship, and a lack of access to trained veterinarians in small farms may facilitate the accumulation of genes on mobile genetic elements which can then further spread in the bacterial population. Finally, incF plasmids comprise the most common plasmid-types among ESBL and/or AmpC-carrying plasmids identified in our data (*n* = 93, 38.6%). These plasmids are known to be highly transmissible among *E. coli*, further stressing the importance of stopping the spread of plasmids that contain genes conferring resistance to extended-spectrum ß-lactams, fluoroquinolones and colistin [71]. IncF plasmids harboring CTX-M-55 and *qnr* have also been reported in other regions of Southeast Asia [70].

### Limitations

Our analysis comes with limitations. First, this analysis focuses on a subset of genomes from ESBL and/or AmpC-producing *E. coli* and thus our results might not be generalizable to the overall population of *E. coli* in Southeast Asia. However, given the importance of these resistance mechanisms in human and animal health and the high prevalence ESBL and/or AmpC (99.4%) on the studied farms, our results provide insights to curb the dissemination of these resistant bacteria. Second, given the lack of information on the quantities of antimicrobials used per class per farm, a full correlation analysis between AMU and ARGs is currently not feasible. Additionally, some farms (*n* = 41, 26.5%) did not report the most common used antimicrobials used. However, our the ARGs picked by GWAS and AcCNET are in agreement with the most commonly used class of antimicrobials per farm size. Third, we did not use long-read sequencing which makes it difficult to recover fully assembled plasmids. Thus, we used MOB-suite to identify contigs that cluster to the same plasmids and identify resistance genes on those plasmids. However, this method may not identify of all contigs belonging to the same plasmid or bin it to different clusters (eg., transmission event IV, Table 3). Therefore, we need to interpret the plasmid sharing with caution. Nonetheless, MOB-suite has shown to be superior performance in recovering plasmids from short-read data compared to other programs to date [72]. Finally, our analysis does not include a temporal component. Therefore, establishing true transmission events (ie., non-colonized host becomes colonized after being in contact with a previously colonized host) is difficult since identifying the same molecular features isolates in two different hosts and/or farms does not exclude the hypothesis of acquisition from an independent source. Longitudinal studies using Bayesian approaches would help better understand the extent of transmission events since they used time-calibrated trees. These methods would also allow to identify the relative contribution and the uncertainty associated with the phylogenetic signal [63].

### Future directions

Our findings highlight the importance of extending global surveillance efforts in rural areas of LMICs where extensive, and intensive animals production systems co-exist [54]. In particular, our work, like others before [13, 73], reassert the need to tailor actions to control AMR according to farm sizes and degree of intensification.

In small farms, better education of the producer and access to veterinary care is important to limit the selection of ARGs conferring resistance to critically important antimicrobials. Therefore, farmers from small-sized farms should have access to trained veterinarians which can help them manage animal health instead of seeking advice on AMU from drug-stores [17]. Moreover, these farmers should also be informed about the negative impact associated with inappropriate antimicrobial use or inadvertently administering them (eg., supplemented feed) on the selection of important ARGs which might lead to loss of productivity, and potentially affect the health of workers [74, 75].

Medium-sized farms should aim to continuously improve biosecurity to reduce AMU. While the data suggests that medium farms have comparatively higher biosecurity standards than small farms, medium farms have higher AMU which could be either due to reported higher disease prevalence [33], or routine use of, and easier access to antimicrobials [19]. This could indicate that the implementation of biosecurity measures is lagging the rapid intensification of farms in this region. Currently, it is challenging to estimate whether additional biosecurity measures could help reduce AMU in medium farms. However, other studies have indicated that the cost in increasing biosecurity is not higher than the cost in AMU if no measures are applied [76].

At the national level, several actions could be undertaken to reduce AMU and thus reduce AMR. First, national surveys of AMU could signal the extent of AMU (mis)-use across Thailand [77]. Second, such data could be used to establish AMU stewardship programs, that potentially include financial penalties [78]. Such program has successfully reduced AMU and improved biosecurity in pig farmers across Danish pig farms. Third, a nation-wide AMR and antimicrobial stewardship educational program should also be undertaken for both farmers and veterinarians since such programs are currently lacking in the curricula of veterinary and animal husbandry courses in Thailand [79]. Such programs should involve both farmers and veterinarians. Farmers should be trained on the best farming practices and encouraged to seek veterinarian advice prior to AMU on farms. Recently, a Thai study indicated that most farmers got advice on health and antimicrobial management through unqualified sources including relatives, other farmers and individuals that might not have veterinarian training [79]. This is frequently the case for small farms that do not have the funds or access to veterinary services and often get advice from drug stores as observed for the farmers in our study [19]. Educational programs in Kenya and Ghana have successfully addressed these issues and made farmers engage more readily with animal healthcare professionals [80–82].

Training of veterinarians on antimicrobial stewardship increases their perceived feasibility of AMU reduction and improves diagnostics leading to a reduction in AMU. While most of the studies were performed in high income countries, farmers and veterinarians in different countries also have different perspectives on AMU and the threat of AMR [83, 84]. Ultimately, adapting many of these programs to the socio-cultural context of Thailand will make all these interventions more successful [53, 85].

Finally, additional studies are needed to understand the environmental sources of AMR and their impact on the maintenance and transmission of AMR bacteria [64]. This would help understand whether the physical proximity to an AMR contaminated environment explains the transmission of resistance between farms, since close proximity we observed for the *bla*_CTX-M-55_-carrying plasmids (Fig. 3A). Such studies might also shed light on the importance of implementing waste management practices that minimize the discharge of antimicrobial residues and/or antimicrobial resistant bacteria into the environment which would promote the maintenance of these bacteria and/or plasmids outside farming environments [63, 64].

## Supplementary Information

**Additional file 1: Table S1. **Thai Study Metadata.

**Additional file 2: Table S2. **ESBL AMPC farm metadata.

## Data Availability

All data generated or analysed during this study is openly available. Genomes’ NCBI BioSample ID and metadata are available in Table S1. R Code and additional files can be found in the following Zenodo repository: https://doi.org/10.5281/zenodo.6807730.
